# A default prior distribution for contingency tables with dependent factor levels

**DOI:** 10.1016/j.stamet.2013.08.007

**Published:** 2014-01

**Authors:** Antony M. Overstall, Ruth King

**Affiliations:** School of Mathematics & Statistics, University of St Andrews, St Andrews, Fife, KY16 9SS, United Kingdom

**Keywords:** Contingency table, Dependence structure, Default prior

## Abstract

A default prior distribution is proposed for the Bayesian analysis of contingency tables. The prior is specified to allow for dependence between levels of the factors. Different dependence structures are considered, including conditional autoregressive and distance correlation structures. To demonstrate the prior distribution, a dataset is considered which involves estimating the number of injecting drug users in the eleven National Health Service board regions of Scotland using an incomplete contingency table where the dependence structure relates to geographical regions.

## 1 Introduction

Contingency tables (e.g.  [1]) are formed when a population is cross-classified according to a series of categories (or factors). Each cell count of the table gives the number observed under each cross-classification. The aim of forming such a table is to summarise the data, and typically, with a view to identifying interactions or relationships between the factors.

The standard statistical practice to model such interactions is the log–linear model (e.g.  [1, Chapter 7]). In this case the logarithm of the expected cell count is proportional to a linear predictor depending on the main effect terms and interaction terms between the factors. Each combination of interaction terms defines its own log–linear model so that the identification of the non-zero interaction terms translates to an exercise in model comparison. Additionally incomplete contingency tables with missing cell counts can be used to estimate closed populations  [4] where some of the factors correspond to sources that have either observed or not observed individuals in the population.

In this paper, we consider the case where the levels of one or more of the factors may be dependent on one another. An obvious example is when one of the factors has levels corresponding to geographical regions or locations which may be dependent due to their geographical proximity. In these cases, we may expect the parameters of the log–linear model to have some dependence structure. Bayesian analysis of contingency tables is common (e.g.  [3], [13], [5]) and is the approach taken here. One feature of the Bayesian approach is that prior information on the interaction terms can be incorporated through the prior distribution. We take the position of having weak prior information on the magnitude of the log–linear parameters but wish to incorporate the information provided by the dependence structure mentioned above. In the case of weak prior information and model uncertainty, care must be taken when specifying prior distributions due to Lindley’s paradox (e.g.  [16, pp. 77–79]). There have been several attempts in the literature (e.g.  [3], [15], [18]) to specify “default” prior distributions that can be applied for log–linear models under model uncertainty. We extend these approaches by developing a default prior that can take account of the dependence structure between the factor levels and can be seen as a generalisation of the above mentioned priors. The proposed prior is constructed by conditioning on the constraints on the parameters which are introduced in contingency table analysis to maintain identifiability of the parameters.

This paper is organised as follows. In Section  2 we set out our notation and briefly describe log–linear models. In Section  3 we derive our proposed default prior distribution including descriptions of different dependence structures. Finally, we apply our proposed prior to a real data application in Section  4, which involves estimating the number of injecting drug users in Scotland. Here, one of the factors corresponds to geographical regions, and we wish to take account of the possible dependence structure that may exist for the regions.

## 2 Notation and log–linear models

### 2.1 Notation

We assume that there are a total of c factors such that each factor k=1,…,c has $l_{k}$ levels. The corresponding contingency table has $n=\prod_{k=1}^{c}l_{k}$ cells. Let y be the n×1 vector of cell counts with elements denoted as $y_{i}$ and where $i=\left(i_{1},\ldots ,i_{c}\right)$ identifies the combination of factor levels that cross-classify the cell i. Let S be set of all n cross-classifications so that

$$S=\left\{\left(i_{1},\ldots ,i_{c}\right):i_{l}\in \left\{1,\ldots ,l_{k}\right\}\right\}\text{.}$$

Finally, let $N=\sum_{i\in S}y_{i}$ be the total population size. In the case of an incomplete contingency table, N is unknown, since elements of y are unknown.

As a pedagogic example that we use for illustrative purposes throughout, suppose that there are three factors used to cross-classify a population of hospital patients: age (2 levels: young; old), hypertension (2 levels: no; yes) and region (3 levels: A; B; C). In this example, c=3, where $l_{1}=2,l_{2}=2$ and $l_{3}=3$, and the three factors (age, hypertension and region) have been labelled 1, 2 and 3, respectively. It follows that there are n=2×2×3=12 cells.

### 2.2 Log–linear models

We now briefly describe log–linear models and initially assume that the form of the log–linear model is known, i.e. it is known which interactions are present. We extend to the case of model uncertainty later in this section. Let $\eta_{i}$ denote the linear predictor associated with cell i∈S, where

$$\eta_{i}=\phi +z_{i}^{T}\theta \text{,}$$

with ϕ∈R denoting the intercept term, θ the q×1 vector of log–linear parameters (i.e. the main effects and interaction terms) and $z_{i}$ the q×1 vector of zeros and ones identifying which elements of θ are applicable to cell i∈S.

For identifiability, certain elements of θ are constrained, e.g. by sum-to-zero, or corner-point constraints, so we can rewrite $\eta_{i}$ as

$$\eta_{i}=\phi +x_{i}^{T}\beta \text{,}$$

where $\beta \in R^{p}$ is the p×1 vector of unconstrained regression parameters, and $x_{i}$ is the p×1 vector which identifies which elements of β correspond to cell i∈S, with p<q.

Finally, let η be the n×1 vector with elements $\eta_{i}$, and let X be the n×p model matrix with rows $x_{i}$. Then we can write

$$\eta =\phi 1_{n}+X\beta \text{,}$$

where $1_{n}$ denotes the n×1 vector of ones.

For the statistical analysis of contingency tables, it is common to assume that

(1) $$y_{i}|\phi ,\beta ∼\text{Poisson}\left(\lambda_{i}\right)\text{,}$$

independently, where $\log\lambda_{i}=\eta_{i}$.

In practice, we typically do not know the form of the log–linear model. This is equivalent to not knowing the elements of $z_{i}$ and $x_{i}$, or the columns of X. Let M be the set of competing log–linear models which are indexed by m∈M. Associated with each log–linear model are $z_{i}^{\left(m\right)},x_{i}^{\left(m\right)},X^{\left(m\right)},\theta^{\left(m\right)}$ and $\beta^{\left(m\right)}$, where $z_{i}^{\left(m\right)}$ and $\theta^{\left(m\right)}$ are $q^{\left(m\right)}\times 1$ vectors, $x_{i}^{\left(m\right)}$ and $\beta^{\left(m\right)}$ are $p^{\left(m\right)}\times 1$ vectors, and $X^{\left(m\right)}$ is an $n\times p^{\left(m\right)}$ matrix.

In the next section, we derive a default prior distribution for $\beta^{\left(m\right)}|m$. For the intercept, ϕ, we assume a prior given by π(ϕ)∝1. Although this prior is improper, the resulting posterior is still proper  [5]. This prior will not cause a problem under Lindley’s paradox since it is present for all models in M[16, p. 174].

## 3 A default prior distribution for $\beta^{\left(m\right)}|m$

### 3.1 Derivation

In this section we develop a default prior distribution for $\beta^{\left(m\right)}|m$. For notational simplicity, we drop the dependency on the model m by removing the superscript (m).

Suppose that there are a total of T log–linear terms and $\beta =\left(\beta_{1},\ldots ,\beta_{T}\right)$ where $\beta_{t}$, for t=1,…,T, is the $p_{t}\times 1$ vector corresponding to the regression parameters for the main effect or interaction term t. Similarly let $\theta_{t}$ denote the corresponding $q_{t}\times 1$ vector of log–linear parameters, for t=1,…,T.

Let $R_{t}$ be the set of f main effect terms that define the f-way interaction $\beta_{t}$. Dellaportas and Forster  [3] refer to $R_{t}$ as the constituent terms of the interaction. Note that $q_{t}=\prod_{j\in R_{t}}q_{j}$ and if $\beta_{t}$ corresponds to a main effect then $R_{t}$ has only one element, i.e. t. Consider the pedagogic example, from Section  2.1, and t corresponding to the 2-way interaction between age and region so that $q_{t}=6$ and $p_{t}=2$. The constituent terms, $R_{t}$, have two elements: the terms corresponding to age and region.

We initially consider deriving the default prior distribution under sum-to-zero constraints. We describe how the prior can be extended to any system of constraints in Section  3.4. Following Dellaportas and Forster  [3] we assume that β has a multivariate normal distribution with mean zero, where $\beta_{r}$ and $\beta_{t}$ are independent for r,t=1,…,T and r≠t. Thus, all that remains is to specify the $p_{t}\times p_{t}$ covariance matrix for each $\beta_{t}$, for t=1,…,T.

The elements of $\theta_{t}$ are subject to constraints and can be written in the form

(2) $$\theta_{t}=A_{t}\beta_{t}\text{,}$$

where $A_{t}$ is a $q_{t}\times p_{t}$ matrix defining the constraints. Under sum-to-zero constraints, $A_{t}$ can be written as

(3) $$A_{t}=P_{t}\left(\begin{array}{c} I_{p_{t}}\\ C_{t} \end{array}\right)\text{,}$$

where $I_{p_{t}}$ is the $p_{t}\times p_{t}$ identity matrix, $C_{t}$ is a $\left(q_{t}-p_{t}\right)\times p_{t}$ matrix and $P_{t}$ is a $q_{t}\times q_{t}$ permutation matrix. For t corresponding to the age and region interaction in the pedagogic example,

$$\theta_{t}=\left(\begin{array}{c} \theta_{t1}\\ \theta_{t2}\\ \theta_{t3}\\ \theta_{t4}\\ \theta_{t5}\\ \theta_{t6} \end{array}\right)\text{,}\;A_{t}=\left(\begin{array}{cc} 1 & 0\\ 0 & 1\\ -1 & -1\\ -1 & 0\\ 0 & -1\\ 1 & 1 \end{array}\right)\text{,}\;C_{t}=\left(\begin{array}{cc} -1 & 0\\ 0 & -1\\ 1 & 1\\ -1 & -1 \end{array}\right)\text{,}$$

$$P_{t}=\left(\begin{array}{cccccc} 1 & 0 & 0 & 0 & 0 & 0\\ 0 & 1 & 0 & 0 & 0 & 0\\ 0 & 0 & 0 & 0 & 0 & 1\\ 0 & 0 & 1 & 0 & 0 & 0\\ 0 & 0 & 0 & 1 & 0 & 0\\ 0 & 0 & 0 & 0 & 1 & 0 \end{array}\right)\text{.}$$

The elements of $\theta_{t}$ are ordered so that the factor levels of region vary the fastest.

Initially, ignoring the constraints that are applied to $\theta_{t}$, we assume that the distribution of $\theta_{t}$ is

$$\theta_{t}|\sigma_{t}^{2},D_{t}∼N\left(0,\sigma_{t}^{2}D_{t}\right)\text{,}$$

where $\sigma_{t}^{2}>0$ and $D_{t}$ is a $q_{t}\times q_{t}$ positive-definite scale matrix. The off-diagonal elements of $D_{t}$ control the dependence structure or correlation between the elements of the constrained parameters, $\theta_{t}$, corresponding to different factor levels.

It follows from (2), (3) that

(4) $$P_{t}^{T}\theta_{t}=\left(\begin{array}{c} \beta_{t}\\ C_{t}\beta_{t} \end{array}\right)\text{.}$$

Let

$$\gamma_{t}=\left(\begin{array}{c} \gamma_{t}^{\left(1\right)}\\ \gamma_{t}^{\left(2\right)} \end{array}\right)=P_{t}^{T}\theta_{t}$$

be the permuted elements of $\theta_{t}$ according to the inverse permutation $P_{t}^{-1}=P_{t}^{T}$, so that $\gamma^{\left(1\right)}=\beta_{t}$ and $\gamma^{\left(2\right)}=C_{t}\beta_{t}$. The prior distribution for $\beta_{t}$ is the conditional distribution of $\gamma^{\left(1\right)}$ (which is $\beta_{t}$) given that $\gamma^{\left(2\right)}=C_{t}\beta_{t}$, i.e. we find the distribution of $\beta_{t}$ from (4) subject to the constraints. It can be shown (see Appendix A) that

(5) $$\beta_{t}|\sigma_{t}^{2},D_{t}∼N\left(0,\sigma_{t}^{2}\Sigma_{t}\right)\text{,}$$

where

(6) $$\Sigma_{t}={\left(A_{t}^{T}D_{t}^{-1}A_{t}\right)}^{-1}\text{.}$$

In the next two sections we consider $D_{t}$. It may be that $D_{t}$ is completely specified *a priori*. The most plausible situation for this is when we assume independence between the levels of this term and $D_{t}=I_{q_{t}}$. We consider this case in Section  3.2. In Section  3.3 we also consider where $D_{t}$ is unknown due to its dependence on some unknown hyperparameter which controls the strength of correlation between the elements of $\theta_{t}$.

### 3.2 Independent correlation structure

Suppose we assume that the factor levels are independent, i.e. $D_{t}=I_{q_{t}}$, so that

$$\Sigma_{t}={\left(A_{t}^{T}A_{t}\right)}^{-1}\text{.}$$

Denote by $X_{t}$ the $n\times p_{t}$ matrix formed by the columns of X corresponding to $\beta_{t}$. Since $X_{t}$ is a permutation of the matrix formed by stacking $A_{t}$ to form an $n\times p_{t}$ matrix, it follows that

$$X_{t}^{T}X_{t}=\frac{n}{q_{t}}A_{t}^{T}A_{t}\text{,}$$

and therefore $\Sigma_{t}=\left(n/q_{t}\right){\left(X_{t}^{T}X\right)}^{-1}$. The corresponding prior distribution for $\beta_{t}$ is

$$\beta_{t}|\sigma_{t}^{2}∼N\left(0,\frac{\sigma_{t}^{2}n}{q_{t}}{\left(X_{t}^{T}X_{t}\right)}^{-1}\right)\text{.}$$

If $\sigma_{t}^{2}=gq_{t}/n$, then since (under sum-to-zero constraints) $X_{t}^{T}X_{r}\neq 0$, for all t≠r   [14], it follows that the prior distribution for $\beta =\left(\beta_{1},\ldots ,\beta_{T}\right)$ is

(7) $$\beta |g∼N\left(0,g{\left(X^{T}X\right)}^{-1}\right)\text{.}$$

If g>0 is unknown and given a prior distribution, then (7) is a hierarchical prior distribution that is identical to the generalised hyper-g prior proposed by Sabanes-Bové and Held  [18] for generalised linear models (GLMs) when applied to log–linear models. If, instead, g is fixed then (7) is the default prior distribution considered by Dellaportas and Forster  [3] who advocate setting g=kn for some constant k, which represents the number of units of prior information. Ntzoufras et al.  [15] use k=1 under their unit information prior for GLMs when applied to log–linear models.

### 3.3 General correlation structure

We now consider terms, t, whose constituent terms, $R_{t}$, contain factors with correlated levels and $D_{t}$ depends on some unknown hyperparameter τ. This hyperparameter, τ, controls the strength of correlation through some structure imposed on $D_{t}$. Initially consider a main effect term t. In this paper we focus on the case where the factor levels correspond to geographical regions or locations and propose two structural forms for $D_{t}$. However there exist many possible applications with correlated factor levels and other correlation structures that can be used depending on the nature of the factor levels.

1. *Conditional autoregressive structure*
Suppose that the $q_{t}$ levels correspond to regions. Let G be the $q_{t}\times q_{t}$ neighbourhood matrix with ijth element
  $$G_{ij}=\left\{ \begin{array}{cc} 1 & \text{if regions }i\neq j\text{ are neighbours ,}\\ 0 & \text{if otherwise ,} \end{array}\right.$$
  for $i,j=1,\ldots ,q_{t}$. Then for the conditional autoregressive (CAR) structure (e.g.  [2]),
  $$D_{t}={\left(I_{q_{t}}-\tau G\right)}^{-1}\text{,}$$
  where τ determines the strength of spatial correlation for the constrained parameters. To ensure that $D_{t}$ is positive-definite, the hyperparameter τ must lie in the interval $\left(\tau_{\min},\tau_{\max}\right)=\left(e_{q_{t}}^{-1},e_{1}^{-1}\right)$, where $e_{1}$ and $e_{q_{t}}$ are the maximum and minimum eigenvalues of G, respectively.
2. *Distance correlation structure*
Suppose the $q_{t}$ levels correspond to locations such as cities. Then the ijth element of $D_{t}$ is given by a correlation function that depends on the distance, $d_{ij}$, between locations i and j, and τ. For example, the Gaussian correlation function gives
  $$D_{t,ij}=\exp\left(-\frac{d_{ij}^{2}}{2\tau^{2}}\right)\text{,}$$
  where, again, τ>0 controls the strength of correlation.

Note that in both examples, the hyperparameter, τ, is not actually a correlation coefficient; it merely controls the strength of correlation. We need to specify a prior distribution for τ. This will depend on the application.

For a term t that corresponds to an interaction term, we propose

(8) $$D_{t}=\underset{r\in R_{t}}{⨂}D_{r}\text{.}$$

The form given by (8) has been chosen for its consistency. Suppose that the correlation between two levels of a main effect term is d. Then, for an interaction involving this main effect, the correlation between the two levels will be d if and only if the factor levels of the other constituent terms are identical. To demonstrate this we return to our pedagogic example where the regions A and B, and B and C are neighbours, but A and C are not neighbours. A CAR structure is specified. In this example, the neighbourhood matrix is

$$G=\left(\begin{array}{ccc} 0 & 1 & 0\\ 1 & 0 & 1\\ 0 & 1 & 0 \end{array}\right)\text{,}$$

so that $D_{t}$ for the main effect of region is

$$D_{\mathrm{region}}=\frac{1}{1-2\tau^{2}}\left(\begin{array}{ccc} 1-\tau^{2} & \tau & \tau^{2}\\ \tau & 1 & \tau \\ \tau^{2} & \tau & 1-\tau^{2} \end{array}\right)\text{.}$$

The eigenvalues of G are $\left(-\sqrt{2},0,\sqrt{2}\right)$, so, therefore, $\tau \in \left(\tau_{\min},\tau_{\max}\right)=\left(-1/\sqrt{2},1/\sqrt{2}\right)$. If an independent correlation structure is specified for the main effect of age, then

(9) $$D_{\mathrm{age}:\mathrm{region}}=\frac{1}{1-2\tau^{2}}\left(\begin{array}{cccccc} 1-\tau^{2} & \tau & \tau^{2} & 0 & 0 & 0\\ \tau & 1 & \tau & 0 & 0 & 0\\ \tau^{2} & \tau & 1-\tau^{2} & 0 & 0 & 0\\ 0 & 0 & 0 & 1-\tau^{2} & \tau & \tau^{2}\\ 0 & 0 & 0 & \tau & 1 & \tau \\ 0 & 0 & 0 & \tau^{2} & \tau & 1-\tau^{2} \end{array}\right)\text{.}$$

The correlation between A and B for the main effect of region is $\tau {\left(1-\tau^{2}\right)}^{-1/2}$. For the age and region interaction, the correlation between levels involving A and B is $\tau {\left(1-\tau^{2}\right)}^{-1/2}$ if and only if they have the same level for age. It now follows from (6), (9) that the scale matrix for the prior distribution is

$$\Sigma_{\mathrm{age}:\mathrm{region}}=\frac{1}{3+4\tau }\left(\begin{array}{cc} 1+\tau & -1/2\\ -1/2 & 1 \end{array}\right)\text{.}$$

If we denote the regression parameters for this term as $\beta_{t}=\left(\beta_{t1},\beta_{t2}\right)$, where t=age:region, then the prior correlation between $\beta_{t1}$ and $\beta_{t2}$ is

$$\mathrm{corr}\left(\beta_{t1},\beta_{t2}\right)=-\frac{1}{2\sqrt{1+\tau }}\text{.}$$

If τ=0, corresponding to independence between the regions, i.e. $D_{t}=I_{q_{t}}$, and thus we have the Sabanes-Bové and Held  [18] prior, then $\mathrm{corr}\left(\beta_{t1},\beta_{t2}\right)=-1/2$. The function $\mathrm{corr}\left(\beta_{t1},\beta_{t2}\right)$ is increasing in τ but the correlation is always negative. This is caused by the sum-to-zero constraints. As τ increases, the magnitude of the negative correlation decreases.

A further advantage of using the structure defined by (8) is computational. If we assume that the independence model, containing only the main effect terms, is the simplest model we wish to consider then we will always have the same set of hyperparameters in each model.

### 3.4 Alternative constraint systems

We now consider alternative constraint systems to sum-to-zero constraints, e.g. corner-point or Helmert constraints. Let $\beta_{A}$ and β denote the vectors of regression parameters under the alternative and sum-to-zero constraints, respectively. Since, under the sum-to-zero constraints, each component, $\beta_{t}$, of β has a normal distribution, then β has a normal distribution with mean zero and variance matrix $\Psi =\mathrm{diag}\left\{\sigma_{1}^{2}\Sigma_{1},\ldots ,\sigma_{T}^{2}\Sigma_{T}\right\}$. It can be shown (see Appendix B) that

(10) $$\beta_{A}={\left(X_{A}^{T}\left(I_{n}-\frac{1}{n}J_{n}\right)X_{A}\right)}^{-1}X_{A}^{T}\left(I_{n}-\frac{1}{n}J_{n}\right)X\beta ,=R_{A}X\beta \text{,}$$

where $X_{A}$ and X are the model matrices under the alternative and sum-to-zero constraints, respectively, $J_{n}$ is the n×n matrix of ones and

$$R_{A}={\left(X_{A}^{T}\left(I_{n}-\frac{1}{n}J_{n}\right)X_{A}\right)}^{-1}X_{A}^{T}\left(I_{n}-\frac{1}{n}J_{n}\right)\text{.}$$

Therefore $\beta_{A}∼N\left(0,\Psi_{A}\right)$, where the prior variance matrix, $\Psi_{A}$, is given by

$$\Psi_{A}=R_{A}X\Psi X^{T}R_{A}^{T}\text{.}$$

Note that, under the alternative constraints, $\beta_{t}$ and $\beta_{r}$ may no longer, necessarily, be independent. This is equivalent to the fact that $\Psi_{A}$ (given by the above expression) may no longer, necessarily, be block diagonal.

Under the independence structure described in Section  3.2, where $D_{t}=I_{q_{t}}$, for t=1,…,T, then

$$\Psi_{A}=gR_{A}X{\left(X^{T}X\right)}^{-1}X^{T}R_{A}^{T}\text{.}$$

The matrix $H=X{\left(X^{T}X\right)}^{-1}X^{T}$ is called the hat matrix and is invariant to the type of constraint system used, i.e. $H=H_{A}=X_{A}{\left(X_{A}^{T}X_{A}\right)}^{-1}X_{A}^{T}$ and therefore

$$\Psi_{A}=g{\left(X_{A}^{T}X_{A}\right)}^{-1}\text{.}$$

Therefore the proposed prior distribution is a generalisation of the default prior distribution of Sabanes-Bové and Held  [18] for any type of constraint system.

## 4 Example: estimating the number of injecting drug users (IDUs) in Scotland from capture–recapture data

**Fig. 1:**
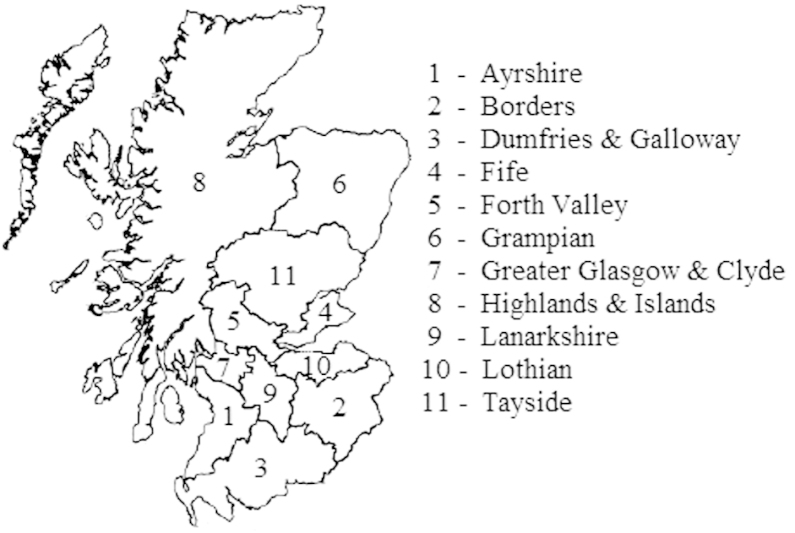
Map showing the eleven regions of Scotland which correspond to National Health Service (NHS) board regions.

In this section we apply our proposed default prior distribution to an incomplete contingency table which has six factors and 352 cells that involves estimating the number of injecting drug users (IDUs) in Scotland in 2006. These data have been previously analysed by King et al.  [12] and Overstall et al.  [17]. The six factors are social enquiry reports (2 levels: observed; unobserved); hospital records (2 levels: observed; unobserved); Scottish drug misuse database (2 levels: observed; unobserved); age (2 levels: ≤35 years; >35 years); gender (2 levels: male; female) and region (11 levels: National Health Service (NHS) board regions—see Fig. 1). The first three factors are sources and the 44 cells which correspond to not being observed by any of these sources for the different age/gender/region combinations have missing counts. Therefore the total population of IDUs, N, is unknown. We use Markov chain Monte Carlo (MCMC) methods to obtain posterior distributions for the missing cell entries and therefore a posterior distribution for the total population of IDUs.

King et al.  [12] and Overstall et al.  [17] merged the eleven regions into just two levels: Greater Glasgow and Clyde, and the Rest of Scotland. Without merging, using all eleven distinct regions, there are small cell counts for many of the regions. For instance, in one region there are only 19 observed IDUs over all source, age and gender cross-classifications. This suggests that a prior distribution that involves smoothing (or borrowing of information), such as the prior proposed in Section  3, is required. We apply the proposed prior where the independence structure is specified for all of the factors except region where we use the CAR structure described in Section  3.3. By calculating the eigenvalues of the neighbourhood matrix, G, for this example, $\tau_{\min}=-0.457$ and $\tau_{\max}=0.247$. We place a uniform prior on τ in the interval $\left(\tau_{\min},\tau_{\max}\right)$. The prior distribution for each $\beta_{t}$ is

$$\beta_{t}|\sigma_{t}^{2},D_{t}∼N\left(0,\sigma_{t}^{2}\Sigma_{t}\right)\text{,}$$

where $\Sigma_{t}$ is given by (6). Following from Section  3.2, we set $\sigma_{t}^{2}=gq_{t}/n$, with

$$g∼\mathrm{IG}\left(\frac{a}{2},\frac{bn}{2}\right)\text{,}$$

where IG denotes the inverse-gamma distribution, and $a=b=10^{-3}$, as suggested by Sabanes-Bové and Held  [18]. We only specify non-zero prior model probabilities for the log–linear models that contain at most two-way interactions and assume a discrete uniform prior over all of these models. It was found that this allowed enough complexity to obtain an adequate overall model when using the Bayesian p-value to assess model adequacy (see,  [8, Chapter 6]).

We use the data-augmentation MCMC approach proposed by King and Brooks  [13] with the reversible jump implementation for GLMs of Forster et al.  [6] to make moves between log–linear models and the weighted least squares Metropolis–Hastings implementation of Gamerman  [7] to make moves within the same log–linear model. We ran the algorithm for one million iterations (discarding the first 10% as burn-in).

For the total population size of IDUs, we obtain a posterior distribution for the total population size with a mean of 21 700 and a 95% highest posterior density interval (HPDI) of (18 900, 24 800). Overstall et al.  [17] obtained a posterior mean of 24 000 and a 95% HPDI of (19 500, 29 700) and King et al.  [12] a mean of 25 000 with a 95% HPDI of (20 700, 35 000). The advantage of our approach over the latter two analyses is that we are able to provide posterior distributions of the total population size in each NHS board region, broken down by age and gender. Our approach also results in a smaller credible interval for the total population size due to it allowing for correlated regions and not discarding information by merging the factor levels of region.

The posterior mean of τ is 0.108 with a 95% HPDI of (−0.096,0.247). The posterior probability of τ being positive is 0.816. It follows that the Bayes factor in support of the hypothesis that τ>0 is 8.205. Therefore there appears to be positive evidence  [11] in support of positive spatial correlation between the regions of Scotland.

## 5 Concluding remarks

In this paper we have proposed a default prior distribution for the regression parameters of a log–linear model that can take account of any dependence structure that may exist between the factor levels. This prior can be applied in situations of model uncertainty and can be seen as a generalisation of other default prior distributions applied to log–linear models including those of Dellaportas and Forster  [3], Ntzoufras et al.  [15] and Sabanes-Bové and Held  [18].

## Appendix A Justification of default prior distribution

In this appendix we give justification for the prior given in Section  3.1, given by (5), (6). The prior distribution for $\beta_{t}$ is the conditional distribution of $\gamma^{\left(1\right)}$ given that $\gamma^{\left(2\right)}=C_{t}\gamma^{\left(1\right)}$, where $\gamma ={\left(\gamma^{\left(1\right)},\gamma^{\left(2\right)}\right)}^{T}∼N\left(0,\sigma_{t}^{2}M\right)$, and $M=P_{t}^{T}D_{t}P_{t}$. Define

$$\psi =\left(\begin{array}{c} \psi^{\left(1\right)}\\ \psi^{\left(2\right)} \end{array}\right)=\left(\begin{array}{cc} I & 0\\ -C_{t} & I \end{array}\right)\left(\begin{array}{c} \gamma^{\left(1\right)}\\ \gamma^{\left(2\right)} \end{array}\right)\text{,}$$

so that we now require the conditional distribution of $\psi^{\left(1\right)}$ given that $\psi^{\left(2\right)}=0$. It follows, from the properties of the multivariate normal distribution, that ψ has a multivariate normal distribution with mean 0 and covariance matrix $\sigma_{t}^{2}T$ where

$$T=\left(\begin{array}{cc} T_{11} & T_{12}\\ T_{21} & T_{22} \end{array}\right)\text{,}$$

and

$$T_{11}=M_{11}\text{,}$$

$$T_{12}=M_{12}-M_{11}C_{t}^{T}\text{,}$$

$$T_{21}=M_{21}-C_{t}M_{11}\text{,}$$

$$T_{22}=M_{22}-M_{21}C_{t}^{T}C_{t}M_{12}+C_{t}M_{11}C_{t}^{T}\text{.}$$

 The partitioning of M and T follows from the partitioning of γ into $\gamma^{\left(1\right)}$ and $\gamma^{\left(2\right)}$. Using the properties of the multivariate normal distribution the covariance matrix of $\beta_{t}$ is $\sigma_{t}^{2}\Sigma_{t}$, where

$$\Sigma_{t}=M_{11}-\left(M_{12}-M_{11}C_{t}^{T}\right){\left(M_{22}-C_{t}M_{12}-M_{21}C_{t}^{T}C_{t}M_{11}C_{t}^{T}\right)}^{-1}\left(M_{21}-C_{t}M_{11}\right)\text{.}$$

Consider the inverse of $\Sigma_{t}$. It can be shown using, e.g.,  [10], and after some matrix algebra, that

$$\Sigma_{t}^{-1}=M_{11}^{-1}+M_{11}^{-1}M_{12}S_{M}^{-1}M_{21}M_{11}^{-1}-M_{11}^{-1}M_{12}S_{M}^{-1}C_{t}-C_{t}^{T}S_{M}^{-1}M_{21}M_{11}^{-1}+C_{t}^{T}S_{M}^{-1}C_{t}\text{,}$$

where $S_{M}=M_{22}-M_{21}M_{11}^{-1}M_{12}$ is the Schur complement (e.g.  [9, p. 95]) of $M_{11}$ in M. As

$$M^{-1}={\left(P_{t}^{T}D_{t}P_{t}\right)}^{-1}=L=\left(\begin{array}{cc} L_{11} & L_{12}\\ L_{21} & L_{22} \end{array}\right)\text{,}$$

then it can be shown that

$$\Sigma_{t}^{-1}=L_{11}+L_{12}C_{t}+L_{21}C_{t}^{T}+C_{t}^{T}L_{22}C_{t},=\left(I\;C_{t}^{T}\right)\left(P_{t}^{T}D_{t}^{-1}P_{t}\right)\left(\begin{array}{c} I\\ C_{t} \end{array}\right),=A_{t}^{T}D_{t}^{-1}A_{t}\text{.}$$

Therefore $\Sigma_{t}={\left(A_{t}^{T}D_{t}^{-1}A_{t}\right)}^{-1}$ as required.

## Appendix B Correspondence of parameters between different constraint systems

In this appendix we provide a justification of the correspondence between the regression parameters under any constraint system and sum-to-zero constraints, given by (10). Let $Z_{A}=\left(1_{n},X_{A}\right)$ and $Z=\left(1_{n},X\right)$ be the n×(p+1) matrices formed by appending the vector of ones to the model matrices under the alternative and sum-to-zero constraints. The vector $\left(\phi_{A},\beta_{A}\right)$, where $\phi_{A}$ is the intercept under the alternative constraints, is given by

$$\left(\begin{array}{c} \phi_{A}\\ \beta_{A} \end{array}\right)={\left(Z_{A}^{T}Z_{A}\right)}^{-1}Z_{A}^{T}Z\left(\begin{array}{c} \phi \\ \beta \end{array}\right),={\left(\begin{array}{cc} n & 1_{n}^{T}X_{A}\\ X_{A}^{T}1_{n} & X_{A}^{T}X_{A} \end{array}\right)}^{-1}\left(\begin{array}{c} n\phi +1_{n}^{T}X\beta \\ \phi X_{A}^{T}1_{n}+X_{A}^{T}X\beta \end{array}\right),=\left(\begin{array}{cc} \frac{1}{n}+\frac{1}{n^{2}}1_{n}^{T}X_{A}U_{A}^{-1}X_{A}^{T}1_{n} & -\frac{1}{n}1_{n}^{T}X_{A}U_{A}^{-1}\\ -\frac{1}{n}U_{A}^{-1}X_{A}^{T}1_{n} & U_{A}^{-1} \end{array}\right)\left(\begin{array}{c} n\phi +1_{n}^{T}X\beta \\ \phi X_{A}^{T}1_{n}+X_{A}^{T}X\beta \end{array}\right)\text{,}$$

where $U_{A}=X_{A}^{T}\left(I_{n}-\frac{1}{n}J_{n}\right)X_{A}$. The expression for $\beta_{A}$, given by (10), easily follows.
